# Biocompatible 3D-Printed Tendon/Ligament Scaffolds Based on Polylactic Acid/Graphite Nanoplatelet Composites

**DOI:** 10.3390/nano13182518

**Published:** 2023-09-08

**Authors:** Magda Silva, Susana Gomes, Cátia Correia, Daniela Peixoto, Adriana Vinhas, Márcia T. Rodrigues, Manuela E. Gomes, José A. Covas, Maria C. Paiva, Natália M. Alves

**Affiliations:** 1 3B’s Research Group, I3Bs—Research Institute on Biomaterials, Biodegradables and Biomimetics, University of Minho Headquarters of the European Institute of Excellence on Tissue Engineering and Regenerative Medicine, Avepark, 4805-017 Guimarães, Portugal; magda.sof.g.silva@gmail.com (M.S.); catia.correia@i3bs.uminho.pt (C.C.); daniela.peixoto@i3bs.uminho.pt (D.P.); adrianavinhas@i3bs.uminho.pt (A.V.); mrodrigues@i3bs.uminho.pt (M.T.R.); megomes@i3bs.uminho.pt (M.E.G.); 2 ICVS/3B’s, Associate PT Government Laboratory, 4710-057 Braga/4805-017 Guimarães, Portugal; 3 Department of Polymer Engineering, Institute for Polymers and Composites, University of Minho, 4800-058 Guimarães, Portugal; su-gomes99@hotmail.com (S.G.); jcovas@dep.uminho.pt (J.A.C.); mcpaiva@dep.uminho.pt (M.C.P.)

**Keywords:** 3D printing, functionalized graphite nanoplatelets, PLA, composites, ligaments, tendons

## Abstract

Three-dimensional (3D) printing technology has become a popular tool to produce complex structures. It has great potential in the regenerative medicine field to produce customizable and reproducible scaffolds with high control of dimensions and porosity. This study was focused on the investigation of new biocompatible and biodegradable 3D-printed scaffolds with suitable mechanical properties to assist tendon and ligament regeneration. Polylactic acid (PLA) scaffolds were reinforced with 0.5 wt.% of functionalized graphite nanoplatelets decorated with silver nanoparticles ((f-EG)+Ag). The functionalization of graphene was carried out to strengthen the interface with the polymer. (f-EG)+Ag exhibited antibacterial properties against *Staphylococcus aureus* (*S. aureus*) and *Escherichia coli* (*E. coli*), an important feature for the healing process and prevention of bacterial infections. The scaffolds’ structure, biodegradation, and mechanical properties were assessed to confirm their suitability for tendon and ligamentregeneration. All scaffolds exhibited surface nanoroughness created during printing, which was increased by the filler presence. The wet state dynamic mechanical analysis proved that the incorporation of reinforcement led to an increase in the storage modulus, compared with neat PLA. The cytotoxicity assays using L929 fibroblasts showed that the scaffolds were biocompatible. The PLA+[(f-EG)+Ag] scaffolds were also loaded with human tendon-derived cells and showed their capability to maintain the tenogenic commitment with an increase in the gene expression of specific tendon/ligament-related markers. The results demonstrate the potential application of these new 3D-printed nanocomposite scaffolds for tendon and ligament regeneration.

## 1. Introduction

In recent years, 3D printing has emerged as tool for the production of complex and personalized products, built layer by layer, and with the advantages of low cost and easy operation [1,2]. Three-dimensional printing finds great potential applications in the medical field, in particular in tissue engineering (TE), to produce scaffolds with complex and reproducible geometries, allowing excellent control of porosity and pore size, which is not possible with traditional manufacturing processes. It also provides the possibility to create customized, patient-specific scaffolds [2,3,4,5]. Fused deposition modeling (FDM) (also known as fused filament fabrication (FFF), is the most commonly used material extrusion 3D printing method, presenting several advantages compared with other techniques [5]. It is reliable and cheap and does not require solvents [6].

Tendon/ligament injuries are some of the most prevailing health problems that affect the adult population worldwide [7]. Various materials and strategies have been suggested to find a scaffold that can help the regeneration of these tissues, restoring its function when it is severely injured [8]. It has been reported that highly porous scaffolds with interconnected pores are essential for nutrient and oxygen diffusion, waste removal, and cell proliferation [3,9]. In addition, the degradation rate of the implant should match the rate of the new tissue formation, allowing it to receive the appropriate level of mechanical load from the scaffold [9,10].

PLA is routinely used for medical applications such as sutures or orthopedic fixation devices [6], being slowly degraded and fully reabsorbed [11]. It is made from bio-based monomers that are obtained from corn or cellulose [6] with easy modulation of its physical and biochemical features by blending with different nanofillers [11]. The mixing or in situ modification with toughening agents, flame retardants, and anti-UV agents have been used to address the unfavorable inherent qualities of PLA (i.e., poor toughness, inflammability, and UV aging) and achieve high-performance PLA composites. However, the addition of these additives may increase the economic cost or even reduce the PLA mechanical properties due their agglomeration [12]. A new bio-based porphyrins approach was suggested by Yang and co-workers [12] to improve PLA’s overall multifunctional performance. The incorporation of only 3 wt.% of vanillin-based porphyrin (VPR) not only enhanced the anti-UV and flame-retardant properties but also enabled significant toughening of the PLA/VPR composites as well as improvements of elongation at break and impact strength. This strategy greatly increases the versatility of PLA composites [12]. Formulations based on PLA have been widely proposed for tendon/ligament regeneration [13,14], but there are very few studies about the use of material extrusion 3D printing for this application [2,15,16], being mainly focused on screw-like scaffolds to help tendon–bone healing after anterior cruciate ligament (ACL) reconstruction [2,16]. This ligament holds a commonly accepted relevance because of the huge number of related injuries and reconstructive surgeries [17].

There has been an increased interest in the use of graphene-based materials such as graphite nanoplatelets (GNPs) and carbon nanotubes (CNTs) for different tissue engineering applications such as cardiac, neural, and tendon/ligament regeneration, mainly focusing their reinforcing effect on polymer composites [7,17]. For instance, Belaid et al. [6] produced 3D-printed scaffolds based on PLA–graphene oxide (GO). Tensile testing demonstrated a 30% increase in the Young’s modulus with the incorporation of 0.3 wt.% GO. Composite scaffolds also promoted bone cell attachment, proliferation, and differentiation [6]. In fact, the nanoscale dimension of graphene matches the cell surface receptors and extracellular matrix (ECM) nanotopography, promoting the adhesion and proliferation of various types of cells. In addition, graphene-based scaffolds may also exhibit additional functional properties such as enhanced electrical conductivity, which may be beneficial for the cellular growth and stimulation of the healing process [7,11]. However, each application involving the addition of graphene in the human body should always be carefully investigated because it was observed to have a size-, shape-, and concentration-time-dependent cytotoxicity [7,11]. It is commonly accepted that using small loadings of graphene materials such as graphene nanoplatelets (composed of single-layer and few-layer graphene) can be effective in the reinforcement of polymeric matrices such as PLA and do not present cytotoxicity [6,11,17,18]. When the polymer matrix is a biomaterial, the possibility of a toxic effect of the fillers is even diminished [19]. Moreover, the functionalization of graphene is of great importance for the scaffold success. The presence of functional groups may enable stronger interactions with the matrix, thus improving simultaneously the reinforcing ability and biocompatibility [7,17].

An effective strategy to reduce the risk of infection is to confer antibacterial properties to the implant [20]. Silver nanoparticles (AgNPs) have been extensively combined with inorganic materials and biopolymers to produce 3D-printed scaffolds with antibacterial activity [21,22].

The objective of this work was the production of 3D-printed scaffolds with controllable dimensions and good mechanical properties to help tendon and ligament regeneration, as well as their *in vitro* investigation. We functionalized the GNPs via a 1,3 dipolar cycloaddition reaction (DCA) of an azomethine ylide, which bonds pyrrolidine groups onto the graphene surface to form amide bonds with the ester groups of PLA under the composite processing conditions [23,24]. Silver nanoparticles were also produced by reducing silver nitrate (AgNO_3_) in dimethylformamide (DMF) and used to decorate the functionalized graphene [25]. A synergetic effect of silver and graphene properties may also occur, such as reported by Kumar S. et al. [26], who produced sheets of reduced GO decorated with silver with improved electrical and antibacterial properties.

Composite filaments based on medical-grade PLA containing a low content (0.5 wt.%) of functionalized and Ag-decorated few-layer graphene were produced and applied to form 3D-printed parts, obtaining porous and reproducible scaffolds. The antibacterial efficiency against Gram-positive and Gram-negative bacteria was confirmed. The mechanical performance and biodegradation of 3D-printed scaffolds were evaluated under physiological conditions, as well as their biocompatibility using L929 cells. The scaffolds were loaded with human tendon-derived stem cells, to investigate the tenogenic commitment and to analyze the gene expression of specific tendon/ligament-related markers. The results are promising and will hopefully widen the application of 3D-printed devices for tissue engineering and demonstrate their potential use in tendon and ligament healing and regeneration.

## 2. Materials and Methods

### 2.1. Materials

Medical-grade PLA pellets (PURASORB^®^ PL10) were purchased from Corbion, Gorinchem, The Netherlands. The PLA exhibited an inherent viscosity in the range 0.9–1.2 dL/g and melting point between 170 and 200 °C. Micrograf HC11, a micronized graphite with a purity of 99.5%, equivalent diameter of approximately 10 µm, and few tens of nanometers of thickness, will be referred, throughout the text, as EG and was purchased from Nacional de Grafite Lda, Itapecerica, MG, Brazil.

EG was functionalized (f-EG) by the DCA reaction as described previously [23] using a functionalization time of 3 h at 250 °C. The decoration of f-EG with silver nanoparticles [(f-EG)+Ag] was achieved by the reduction reaction of silver ions (Ag^+^). The detailed procedures and characterization were previously described [23].

### 2.2. Antimicrobial Potential of Functionalized Graphite

The antibacterial properties of different graphite nanoplatelets (EG, f-EG, and [(f-EG)+Ag]) were evaluated directly against microorganisms of clinical relevance, namely, Gram-positive *Staphylococcus aureus* (*S. aureus*) (ATCC 25923) and Gram-negative *Escherichia coli* (*E.coli*) (ATCC 25922). First, *E. coli* and *S. aureus* strains were cultured on Mueller Hinton Broth (MHB) at 37 °C and 60 rpm, and the microbial suspension was adjusted to 1.0 × 10^6^ CFU/mL. Then, different EGs were sterilized using 1 h of UV lights and dispersed in a sterilized MHB medium to obtain a concentration of 2% *w*/*v*. The different EG and EG derivatives suspensions were sonicated 1 h before the assay, and then several dilutions were prepared for the tests (1.00, 0.50, 0.25, 0.10, 0.050, and 0.025% *w*/*v*).

In a 96-well plate, 50 μL of the bacterial suspension was added to 50 μL of the different concentrations of EGs suspensions. The plate was incubated at 37 °C for 24 h. Then, aliquots from each well (10 μL) were added onto the surface of nutrient Mueller Hinton agar (MHA) and incubated at 37 °C for 24 h. The minimal bactericidal concentration (MBC) was determined as the lowest concentration that showed no bacterial growth on the agar plate. Several controls were used: a bacterial suspension without EGs, a bacterial suspension with Kanamycine (5% *w*/*v*), and different EGs suspensions without a bacterial suspension. All assays were performed in triplicate. The obtained results allowed the selection of the percentage of EGs for composites’ preparation.

### 2.3. Filaments Production and Characterization

According to the literature, low concentrations of GNPs did not present *in vitro* cytotoxicity and may be incorporated safely in PLA to improve aspects relevant for biomedical applications, such as mechanical properties [18,27]. Based on this consideration and the antibacterial results, we fixed the filler content at 0.5 wt.%. To ensure a good dispersion of the reinforcements in the polymer, a pre-mixing of PLA pellets with 0.5 wt.% of EG, f-EG, and (f-EG)+Ag was prepared by manual mixing. The PLA and PLA composite filaments were obtained by using melt extrusion on a co-rotating twin-screw extruder (Microlab Rondol, Nancy, France) equipped with intermeshing screws containing three mixing zones, using a screw speed of 43 rpm and a temperature profile 135/185/≃160 °C (feed/barrel/die). The extrudate diameter was controlled using two pulling rolls, distanced approximately 25 and 60 cm from the shaping die, respectively. The extrusion process followed the procedure described before [23] under nitrogen atmosphere, as recommended by the polymer manufacturer, to minimize polymer degradation. The detailed processing conditions for each material are presented in Table S1, where pulling roll 1 was located next to the extruder die and allowed the production of filaments with approximately 1.75 ± 0.25 mm, suitable for FDM.

Scanning electron microscopy and energy dispersive spectroscopy (SEM/EDS) were carried out on a FEI Nova 200 FEG-SEM/EDS (FEI Europe Company, Hillsboro, OR, USA) to analyze the coating of the PLA pellets with EG and EG derivatives and to observe the cross-sections of the 3D printing filaments produced. The melt flow index (MFI) of all filaments was measured at 190 °C using a load of 2.16 kg on MFI equipment from Daventest (Welwyn Garden City, UK).

High-definition Kelvin force microscopy (HD-KFM) was used to assess the surface electric potential of the PLA+0.5[(f-EG)+Ag] filament, using a Nano-Observer AFM microscope, CSInstruments (Les Ulis, France). The measurements were carried out on longitudinal sections of composite filaments (10 × 10 μm), using a 1V AC signal, at 53 KHz applied to the surface.

### 2.4. Scaffolds Production and Characterization

The 3D-printed scaffolds were designed using the Ultimaker Cura (version 4.4, Ultimaker, Geldermalsen, The Netherlands) software and printed horizontally using an Ender-3 3D Printer from Creality (London, UK). The printing parameters are given in Table 1. The scaffolds exhibited a cylindrical shape with a full length of approximately 32 mm and a diameter of 9 mm, which were comparable to the dimensions of the native ACL. Smaller specimens with an approximate diameter of 9 mm and 4 mm of thickness were also produced for further testing.

#### 2.4.1. Physical and Morphological Analysis of Scaffolds

The scaffolds’ morphology as well as the pore size and distribution were analyzed using a Leica DMS1000 digital microscope (Wetzlar, Germany). The average pore size for each scaffold was obtained by running Image J software (version 1.52, National Institutes of Health, Bethesda, MA, USA) on the digital microscopy images. Perpendicular lines at 15 pores from one border of the pore toward the other were measured by the software, at the top, front, and lateral views of the scaffold. Each measurement was taken using the green channel and with improved contrast.

The porosity of the as-prepared 3D scaffolds was determined using the liquid displacement method similar to that reported by Guan et al. [28] and Zhang and Ma [29]. Ethanol was chosen as a displacement liquid because it could permeate through the porous scaffolds and did not induce swelling or shrinking of the material. Each scaffold was immersed in a cylinder containing a known volume of ethanol (*V*1). The sample was kept soaked in ethanol for 5 min. Then, the ethanol was pressed to force air from the scaffold and to penetrate and fill the pores. The total volume of ethanol and the ethanol-impregnated scaffold was recorded as *V*2. The ethanol-impregnated scaffold was removed from the cylinder, and the residual ethanol volume was recorded as *V*3. The porosity of the scaffold (%) was given by

(1) $$Porosity\;\left(\%\right)=\left(\frac{V1-V3}{V2-V3}\right)\times 100.$$

The average of three measurements was taken for each sample. To confirm a homogenous dispersion of EG and EG derivatives on the PLA, the 3D-printed scaffolds were cryo-fractured, and the scaffold cross-sections were sputter-coated with gold and observed by using SEM/EDS on a FEI Nova 200 FEG-SEM/EDS (FEI Europe Company, Hillsboro, OR, USA).

The topography and roughness of the PLA and PLA+[(f-EG)+Ag] scaffolds were determined at the outer layer of the measured scaffolds. The measurements were performed by using a Nano-Observer AFM, CSInstruments (Les Ulis, France), and the operation mode was oscillating, with an amplitude of 5V and automatic frequency around 60 KHz. AFM topography images with dimension (10 × 10) μm^2^ were obtained. The root mean square (RMS) surface roughness was calculated by using the statistical tool in the Gwyddion software. This represented the standard deviation of the distribution of surface heights, and it was more sensitive than the arithmetic average height (Ra) to large deviation from the mean line. At least three measurements were performed for each type of scaffold.

#### 2.4.2. Mechanical/Viscoelastic Properties

The dynamical mechanical analysis (DMA) was carried out to evaluate the mechanical performance and viscoelastic properties of the scaffolds subjected to cyclic loading and immersed in physiologic fluids. The scaffolds were previously soaked overnight in a PBS solution at 37 °C. The DMA analysis was performed using TRITEC2000B equipment (Triton Technology, Grantham, UK) in the compressive mode. The DMA spectra were obtained at the same temperature, applying cycles of increasing frequency from 0.1 to 16 Hz. At least three samples were tested for each composition and scaffold type.

#### 2.4.3. Biodegradation

The degradation rates of different scaffolds (9 mm of diameter and 4 mm of thickness) were evaluated *in vitro* by measuring their initial weight and soaking them into a phosphate buffered saline (PBS, pH = 7.4, Sigma-Aldrich, Saint Louis, MO, USA) at 37 °C. The PBS solution was changed every 3 days. At predefined periods (15 days, 6 weeks, and 12 weeks) the samples were removed from the solution, washed with distilled water to remove the excess salts, and dried at 37 °C for 2 days. The mass loss was calculated using the following equation:

(2) $$Weight\;loss\;\left(\%\right)=\left(\frac{M_{i}-M_{f}}{M_{i}}\right)\times 100$$

where *M_i_* and *M_f_* are the weights of the scaffolds before and after degradation, respectively. Three replicates per composition were analyzed, and the results are presented as an averaged value ± standard deviation.

Specimens of PLA and PLA+0.5EG/f-EG/[(f-EG)+Ag] were also observed by SEM and compared with images of non-degraded samples, to identify the surface erosion after 12 weeks of hydrolytic degradation. To evaluate the changes in the mechanical properties after the complete degradation period, the scaffolds (*n* = 3 or 4 per scaffold composition per time point) were immersed in 1 mL of PBS (pH = 7.4) at 37 °C overnight and tested by dynamical mechanical analysis, using the method described above.

#### 2.4.4. Biological Assays—L929 Cell Line

**Cell seeding.** For the *in vitro* cell studies, L929 mouse fibroblast-like cells (NCTC clone 929, ATCC^®^ CCL-1™, acquired from ATCC^®^ (Manassas, VA, USA), passage P26) were cultured in Dulbecco’s modified minimum essential medium (low glucose DMEM, Sigma-Aldrich, Saint Louis, MO, USA) supplemented with 10% fetal bovine serum (FBS, Life Technologies, Paisley, UK) and 1% of an Antibiotic-Antimycotic (A/A) solution (Life Technologies, Paisley, UK), in 150 cm^2^ tissue culture flasks. The cells were maintained in a humidified air atmosphere containing 5% CO_2_ at 37 °C to grow, and the medium was replaced every 3 days until a 90% confluence was reached. Then, the cells were washed with Dulbecco’s phosphate buffered saline (DPBS, Life technologies, Carlsbad, CA, USA) and detached with 5 mL of trypLE™ express solution (Life technologies, Paisley, UK) for 5 min at 37 °C. An amount of 10 mL of culture medium was added to inactivate the trypLE™. The cells were centrifuged at 300 rcf for 5 min, and the obtained pellet was resuspended in the culture medium.

Before the cell seeding, the scaffolds (diameter = 9 mm, thickness = 4 mm) were sterilized by immersion in 70% ethanol (*v*/*v*) for 1 h and by exposition to UV light, for 30 min on both sides.

The sterile scaffolds were placed in a 24-wells suspension culture plate, and 200 µL of a cell suspension in DMEM culture medium (2 × 10^5^) was added to each well. The samples were then incubated at 37 °C in a humidified air atmosphere of 5% CO_2_. After 4 h of seeding, fresh culture medium was added to each well until reaching 1 mL of volume. The seeding procedure was also applied on tissue culture polystyrene (TCPS, Sarstedt, Singapore) to be used as a positive control.

**Live/Dead staining.** The viability of the L929 cells was evaluated by Calcein AM (ThermoFisher Scientific, Bleiswijk, The Netherlands) and Propidium Iodide (PI) (ThermoFisher Scientific, Bleiswijk, The Netherlands) staining. Before staining, at each time point (1, 3, 7, and 14 days of culture), the culture medium was removed, and each sample was immersed with 1 mL of DMEM medium supplemented with 2 μg Calcein AM and 1 μg PI, for 30 min, in the dark. After that, the samples were washed with PBS and analyzed using an inverted confocal microscope with incubation (TCS SP8, Leica, Germany). All experiments were performed in triplicate.

**SEM.** The attachment and morphology of L929 cells were analyzed by SEM. The scaffolds were removed from the wells after 1, 3, 7, and 14 days of culture; washed with PBS; and then fixed with 2.5% glutaraldehyde (Sigma-Aldrich, Saint Louis, MO, USA) for 2 h. After dehydration in a graded series of ethanol (50%, 70%, 80%, 90%, and 100%) and thermostatic drying, the scaffolds were gold sputtered for analysis.

**DAPI-Phalloidin staining.** The morphology and cytoskeletal organization of the cells was visualized by fluorescent microscopy after staining with phalloidin tetramethylrhodamine and 4′,6-diami-dino-2-phenylindole (DAPI, Sigma-Aldrich, Saint Louis, MO, USA). After 1, 3, 7, and 14 days of culture, the medium was removed and washed with PBS fixed by using neutral buffered formalin (10%, ThermoFisher Scientific, Waltham, MA, USA) for 30 min. Then, the fixed samples were permeabilized using Triton X-100 (0.2% *v*/*v* in PBS, Sigma-Aldrich, Saint Louis, MO, USA) for 5 min and immersed in bovine serum albumin (BSA, 3% *w*/*v* in PBS, Sigma-Aldrich, Saint Louis, MO, USA) for 30 min. The seeded scaffolds were stained with DAPI (1:1000 in PBS, pH = 7.4) for 5 min and rhodamine-phalloidin (1:100 in PBS, pH = 7.4, Sigma Aldrich, Saint Louis, MO, USA) for 30 min. The samples were extensively washed with PBS and analyzed using an AiryScan 2 confocal microscope (LSM 980, Zeiss, Germany).

**Alamar blue.** The metabolic activity of the cells was determined by using the Alamar blue method for 1, 3, 7, and 14 days of culture. After each time point, the culture medium was removed, and a fresh medium supplemented with 20% of Alamar blue reagent (Bio-Rad, Hercules, CA, USA) was added to the cultured scaffolds. The samples were incubated in the dark, for 4 h, at 37 °C, in a humidified air atmosphere of 5% CO_2_. Following this, 100 μL of each solution was transferred to a 96-well black plate to measure the fluorescence at the 590 nm emission wavelength and the 530 nm excitation wavelength using a microplate reader (BIO-TEK Instruments, Winooski, VT, USA).

#### 2.4.5. Biological Assays—Human Tendon-Derived Cells

**Human tendon-derived cells (hTDCs)**—**Isolation and Culture.** The hTDCs were isolated from tendon surplus samples under established protocols with Hospital da Prelada (Porto, Portugal). The samples were provided with the informed consent of the patients, and the procedures were reviewed and approved by the Hospital Ethics Committee (P.I. Nº. 005/2019). The hTDCs were isolated and cultured as previously described [30,31,32,33]. First, the tissue explants were dropwise rinsed in a sterile solution of PBS. The excess of PBS was eliminated using a filtration system for 50 mL tubes (Falcon, Shawnee, USA). The tissue samples were mechanically minced and placed into a 50 mL tube with an enzymatic solution consisting of collagenase (0.1%, Sigma-Aldrich, C6885, Saint Louis, MO, USA), 2 M CaCl_2_ (1:1000, VWR, Darmstadt, Germany), and 1% BSA (Sigma-Aldrich, Saint Louis, MO, USA), followed by a 1 h incubation at 37 °C under agitation. The digested tissue was centrifuged three times at 290 g for 5 min. The pellet of hTDCs was then expanded in a complete culture medium consisting of a medium essential alpha (α-MEM, Invitrogen, Life Technologies Limited, Paisley, UK) supplemented with 10% FBS (Life Technologies) and 1% A/A in humidified 5% CO_2_ atmosphere and used at passage 1–3.

**Cell culture on PLA and PLA+0.5[(f-EG)+Ag] scaffolds.** The hTDCs were seeded at a density of 1.2 × 10^5^ cells per scaffold and cultured in α-MEM medium for 7 and 14 days in humidified 5% CO_2_ atmosphere. Two experimental conditions were considered in which the hTDCs were seeded on (i) PLA scaffolds or (ii) PLA+0.5[(f-EG)+Ag] scaffolds to investigate the potential of these scaffolds for tendon/ligament applications. The hTDCs’ response was investigated by assessing tendon-associated markers at the gene and protein levels.

**RNA Extraction and Real-Time RT-qPCR.** The total ribonucleic acid (RNA) was extracted using the RNeasy Mini Kit (Qiagen, Hilden, Germany) following the manufacturer’s instructions and was quantified using a Nanodrop^®^ ND-1000 spectrophotometer (ThermoFisher Scientific, Wilmington, NC, USA) at 260/280 nm. Complementary DNA was synthesized from 1 μg of RNA of each sample using a qScript^TM^ cDNA Synthesis Kit (Quanta Biosciences, Gaithersburg, MD, USA) according to the manufacturer’s protocol using a Mastercycler^®^ Realplex (Eppendorf, Hamburg, Germany). The transcripts quantification presented in Table S2 was carried out via quantitative polymerase chain reaction (qPCR) using the PerfeCTA SYBR Green FastMix Kit (Quanta Biosciences, Gaithersburg, MD, USA) according to the kit instructions in a Real-Time Mastercycler ep realplex thermocycler (Eppendorf, Hamburg, Germany). Glyceraldehyde-3-phosphate dehydrogenase (GAPDH) was used as reference genes. The relative expression level was calculated using the 2^−ΔΔCt^ method for each target gene [34].

**Immunofluorescence of tendon-related markers in 3D scaffolds.** The hTDCs cultured on the 3D scaffolds were fixed with 10% (*v*/*v*) neutral buffered formalin (ThermoFisher Scientific, Waltham, MA, USA) and permeabilized with 0.025% (*v*/*v*) Triton X-100 (Sigma-Aldrich, Saint Louis, MO, USA) in PBS for 10 min. Afterward, the samples were washed three times with PBS, blocked with Normal Horse Serum (RTU Vectastin Kit, PK-7200, Vector, Burlingame, CA, USA), and incubated overnight at 4 °C with antibodies against Tenomodulin (TNMD, Rabbit anti-human, ab81328, 1:100, Abcam, Cambridge, UK), Scleraxis (SCX, Rabbit polyclonal anti-SCX, ab58655, 1:100, Abcam, Cambridge, UK), and Collagen type I (COL1, Rabbit polyclonal anti-COL1, 47972, 1:100, Novus Biologicals^TM^, ThermoFisher Scientific, Waltham, MA, USA). Subsequently, the samples were washed in PBS and incubated with anti-rabbit Alexa Fluor 488-fluorescent secondary antibody or anti-rabbit Alexa Fluor 594 antibodies (ThermoFisher Scientific, Waltham, MA, USA) for 1 h at RT. All antibodies were diluted in 0.1% BSA/PBS. The samples were rinsed with PBS and stained with 4,6-Diamidino-2-phenyindole, dilactate (DAPI, 5 mg/mL, D9564, Sigma-Aldrich, Saint Louis, MO, USA) for 10 min. The immunolabeled samples were observed by confocal laser scanning microscopy (CLSM, TCS SP8, Leica, Wetzlar, Germany).

### 2.5. Statistical Analysis

The presented data were expressed as the mean ± standard deviation (SD) of at least three replicates, except for RT-PCR analysis, which was expressed as the mean ± standard error of the mean (SEM) of two independent experiments (*n* = 2). The error bars presented in the graphs denote the SD. The statistical analysis was performed using the GraphPad Prism6 software from Windows. The statistical significance was evaluated by one-way ANOVA after performing the Shapiro–Wilk test for normal distribution and by two-away ANOVA followed by Bonferroni post hoc test multiple comparison tests for RT-PCR. A difference was considered significant with a confidence interval of 95% for different degrees of confidence, *p* < 0.05 (*), *p* < 0.01 (**), *p* < 0.001 (***), and *p* < 0.0001 (****).

## 3. Results and Discussion

### 3.1. Antimicrobial Potential

The potential antimicrobial activity of EG and EG derivatives against *E. coli* and *S. aureus* was tested, and the results obtained after 24 h are presented in Table 2. Soft agar plates incubated with the suspensions of bacteria and different concentrations of EG and EG derivatives observed after 24 h, as well as positive and negative controls, are shown in Figure S1.

Bacterial colonies of both *E. coli* and *S. aureus* were found in the presence of EG and f-EG, at different concentrations. However, no bacterial growth of *S. aureus* or *E. coli* was observed for 0.25 up to 1% of (f-EG)+Ag. The MBC of (f-EG)+Ag was 0.25% for *S. aureus*, while for *E. coli* it was only 0.1%. These results provided evidence that the presence of silver nanoparticles conferred antimicrobial properties against these two bacteria strains. This is in agreement with the literature that reports the antibacterial efficacy of silver-containing materials such as PLA fibrous membranes for tendon repair [35] and even composite mats of PLA-GO with antibacterial effects against *E. coli* and *S. aureus* [36]. Although the mechanism of the antibacterial action of AgNPs is not fully understood, it is suggested that silver ions could interact with bacterial cells in several ways [20], such as disturbing the permeability of the cells wall or even penetrating them, causing damage and changing the microbial DNA and proteins [20].

### 3.2. Filaments’ Production and Characterization

A pre-coating of the PLA pellets with EG and EG derivatives was applied before the filament extrusion process. Figure S2 shows a uniform distribution of the EG flakes onto the surface of the PLA pellets. The filament cross-sections were observed by using SEM (Figure S3a–d) and revealed a homogenous dispersion of the fillers in the PLA matrix. The MFI of the filaments was measured to evaluate their potential printability. The MFI values were similar for the PLA and composite filaments, ranging from 24 to 26 g/10 min, which were adequate for use in 3D printing (Figure S3e) [37].

**Figure 1:**
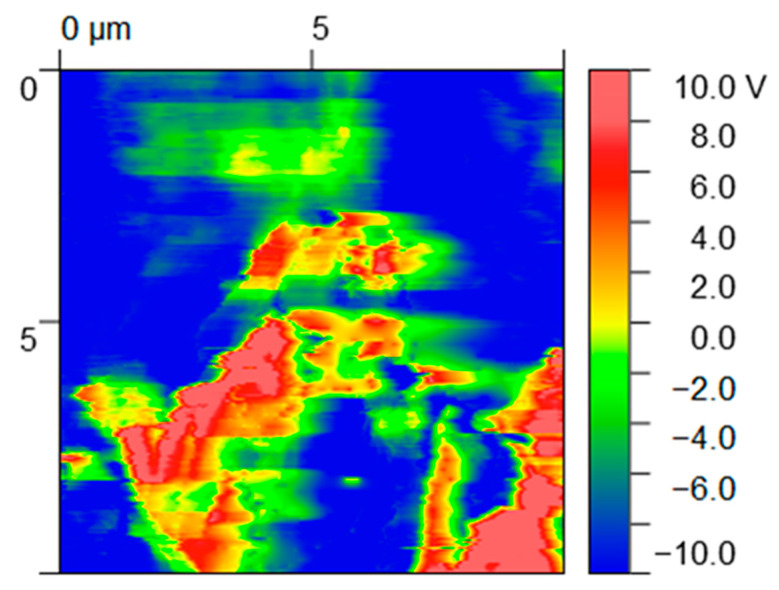
Maps of surface potential (V) obtained from HDKFM potential for composites containing PLA+0.5[(f-EG)+Ag].

The incorporation of conductive particles in a polymer matrix reduces the electrical resistivity of the latter and allows electronic transport/mobility. Considering the envisaged application, it may have a positive effect on the cellular adhesion and growth [38,39]. In addition, the rate of wound healing *in vivo* is closely correlated with changes in the electrical current generated from the wound site [40,41]. We used high-definition Kelvin force microscopy to obtain a map of the variation in the surface potential of the PLA+0.5[(f-EG)+Ag] filaments (Figure 1).

EG and EG derivatives are conductive particles (red regions) and thus show a contrasting surface potential relative to the polymer matrix (dark blue regions), presenting high relative potential values (10 V) [42]. A charge outflow from the graphene flakes can be seen around these particles since EGs have low electron affinity, as observed in other works [42].

### 3.3. Scaffolds’ Characterization

#### 3.3.1. Physical and Morphological Analysis

**Figure 2:**
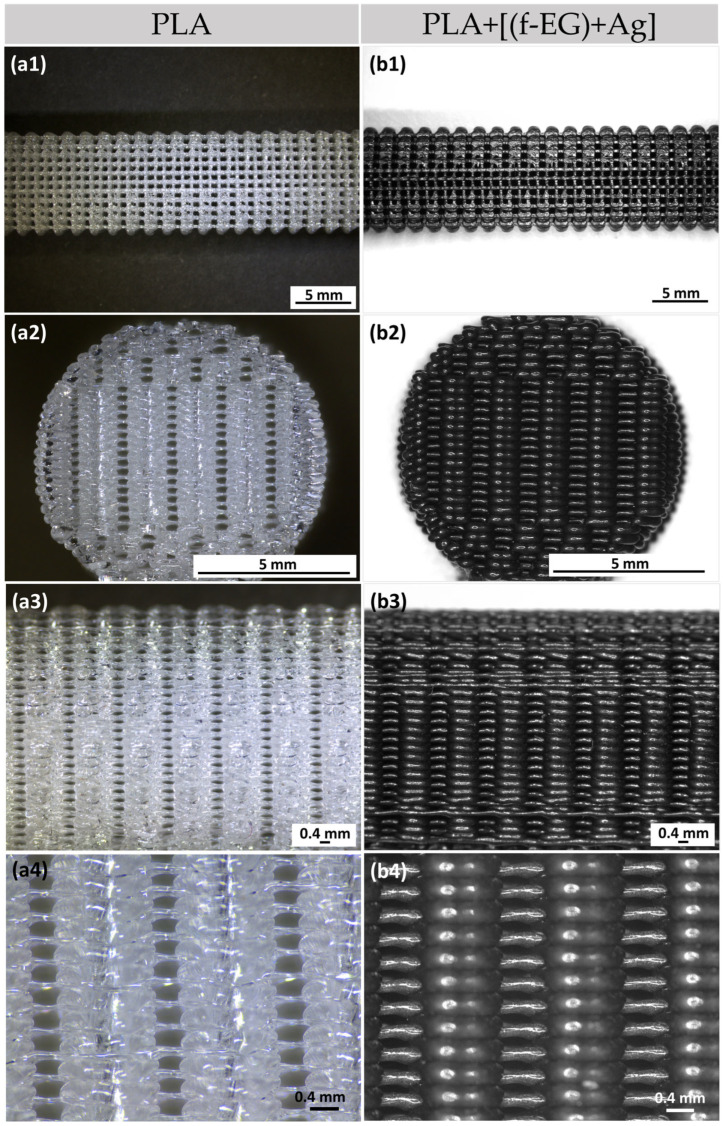
Three-dimensional-printed scaffolds of (**a1**–**a3**) PLA and (**b1**–**b3**) PLA+0.5[(f-EG)+Ag] from the top, front, and side views. Magnification of the (**a4**) PLA and (**b4**) PLA+0.5[(f-EG)+Ag] scaffold structure.

The 3D-printing process was easy to set up and fast to carry out, taking 7 min to print a scaffold with dimensions equivalent to those of the average ACL. This process yielded reproducible samples with high structural homogeneity and controlled geometry, indicating that the printing parameters could be easily adjusted according to the shape and location of the injured tissue [43]. Figure 2 illustrates the 3D-printed PLA (a1–a4) and PLA+0.5[(f-EG)+Ag] scaffolds (b1–b4), at the top, front, and lateral views, at increasing magnifications of their porous morphology.

**Figure 3:**
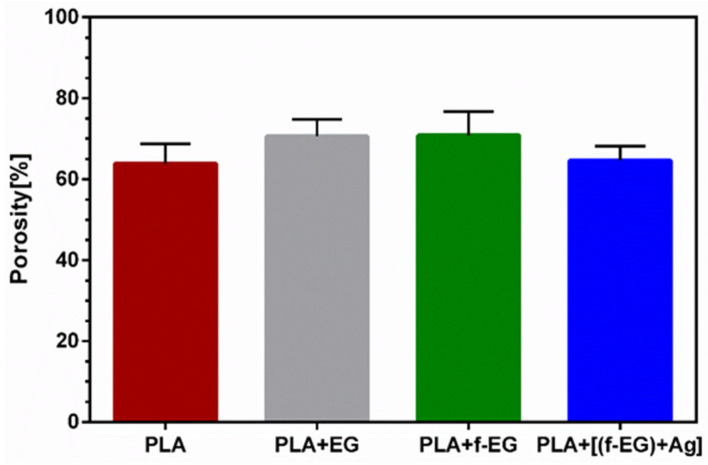
Mean porosity of 3D-printed scaffolds obtained by the liquid displacement method.

The 3D-printed scaffolds presented well-defined pores and interconnectivity. The pore sizes of the PLA and PLA+[(f-EG)+Ag] scaffolds were similar: 430 ± 130 μm and 430 ± 0.100 μm, respectively. Comparable pore sizes were found for scaffolds with other compositions, with no statistically significant differences (*p* < 0.05) (Table S3). The porosity of all scaffolds was measured by the liquid displacement method yielding results from 64 to 71% as observed in Figure 3, with no significant impact caused by the presence of EGs.

The scaffolds for tendon/ligament regeneration should offer a high porosity (ranging from 50 to 85%) and large interconnected pores, with diameters ranging from approximately 250 to 500 μm, to enable cell ingrowth and the flow of nutrients and waste products [44,45]. Thus, the general structural parameters found in the 3D-printed scaffolds enable their use in cell seeding. The scaffold morphologies obtained in this study were in accordance with other studies about 3D-printed scaffolds filled with carbon-based materials, which reported pore sizes ranging from approximately 300 to 500 μm [6,46].

Collagen (Col), silk, PLA, polycaprolactone (PCL), polyglycolic acid (PGA), and polylactide-co-glycolide (PLGA), as well as their composites, have been used as scaffold materials for tendon/ligament replacements, mainly in the form of fibrous scaffolds engineered with textile-based techniques [47]. Different solutions have demonstrated adequate mechanical properties, as well as the ability to sustain cell adhesion and proliferation under satisfactory conditions [14,48,49]. For instance, Sahoo et al. [50] produced a complex hybrid scaffold system by coating basic fibroblast growth factor (bFGF)-releasing PLGA fibers onto the surfaces of a knitted silk scaffold. Rabbit bone-marrow-derived mesenchymal stem cells grew on PLGA fibers and silk microfibers and exhibited good viability. The release of bFGF stimulated cell proliferation and the gene expression of tendon/ligament-specific ECM proteins increased the collagen production and, hence, the mechanical properties of the scaffold [50]. Despite the significant advancements in the tendon/ligament TE, the current solutions have not yet reached the clinic or even the pre-clinic due to some drawbacks in the application including poor mechanical strength and quick degradability or insufficient biological activity [7]. Another remaining problem is the lack of reproducibility and ability to precisely control the pore size and interconnectivity, as well as the scaffolds’ structure and mechanical properties [47]. Some of the major advantages of the proposed scaffolds are their simplicity and controlled architecture and porosity. The scaffolds’ polymer matrix based on PLA provides the possibility to produce scaffolds easily and cost-effectively by 3D printing. The 3D technology was also suggested by Jiang et al. [15] to produce PLGA scaffolds with collagen-fibronectin hydrogels for rotator cuff tendon regeneration. This composite scaffold promoted the proliferation and tenogenic differentiation of human adipose stem cells (ADSCs).

**Figure 4:**
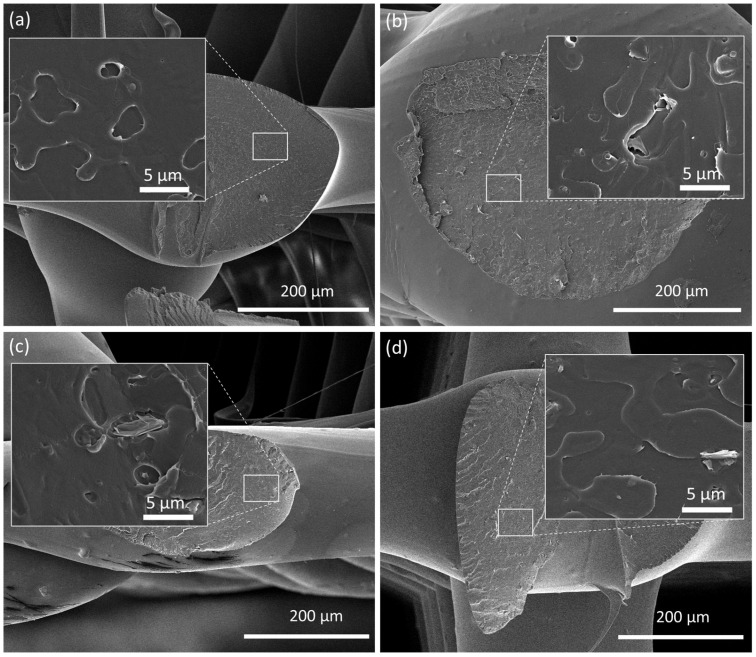
SEM images of the cross-section of 3D-printed scaffolds formed by (**a**) PLA, (**b**) PLA+0.5EG, (**c**) PLA+0.5f-EG, and (**d**) PLA+0.5[(f-EG)+Ag]. The insets represent a higher magnification.

The cryo-fractured cross-sections of all 3D-printed scaffolds were observed by SEM, as illustrated in Figure 4 and Figure S4, to evaluate if the re-melting of composite filaments led to the re-agglomeration of EG. The presence of silver nanoparticles was confirmed by EDS analysis (Figure S5). The obtained images illustrate a homogenous dispersion of EGs after 3D printing. When well dispersed, the large surface area of EGs maximizes the interfacial area, which results in an enhanced load transfer ability [7]. The smoothness of the external surface can also be observed, the composite scaffolds showing a slightly rougher surface due to the EG nanoparticles.

**Figure 5:**
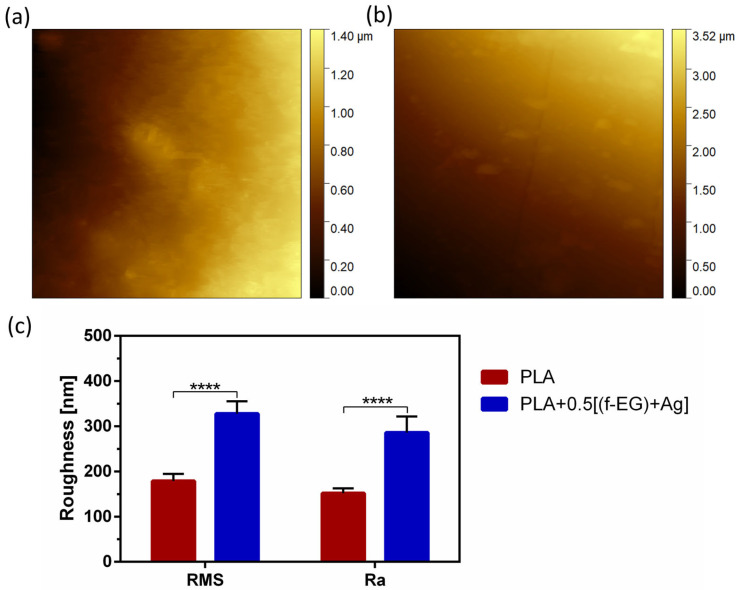
Surface topography of (**a**) PLA and (**b**) PLA+0.5[(f-EG)+Ag] scaffolds as well as (**c**) RMS and Ra of both scaffolds. Significant differences were stated for *p* < 0.0001 (****).

The surface morphology of the 3D-printed PLA and PLA+0.5[(f-EG)+Ag] scaffolds was analyzed by AFM (Figure 5) and SEM (Figure S6a,d), respectively. The average roughness (Ra) and the root-mean-square (RMS) of the surfaces are represented in Figure 5c.

Both scaffolds exhibited a surface roughness at the nanoscale, which increased with the presence of (f-EG)+Ag. In consonance with these observations, the composite scaffolds also presented higher Ra values. This was consistent with the observations of other works concerning 3D-printed PLA scaffolds reinforced with GO [6] and films of PLA reinforced with GNPs [27]. Nanoscale topography has been receiving great attention because of its potential to influence cellular response and its similarity to *in vivo* surroundings [38]. As a comparison, Wu et.al [51] used a coating of PLGA fibers on PLA microfiber yarns to provide topological cues to guide the behavior of human ADSCs in terms of proliferation, migration, collagen secretion, and tenogenic differentiation [51].

#### 3.3.2. Mechanical Properties of Scaffolds

**Figure 6:**
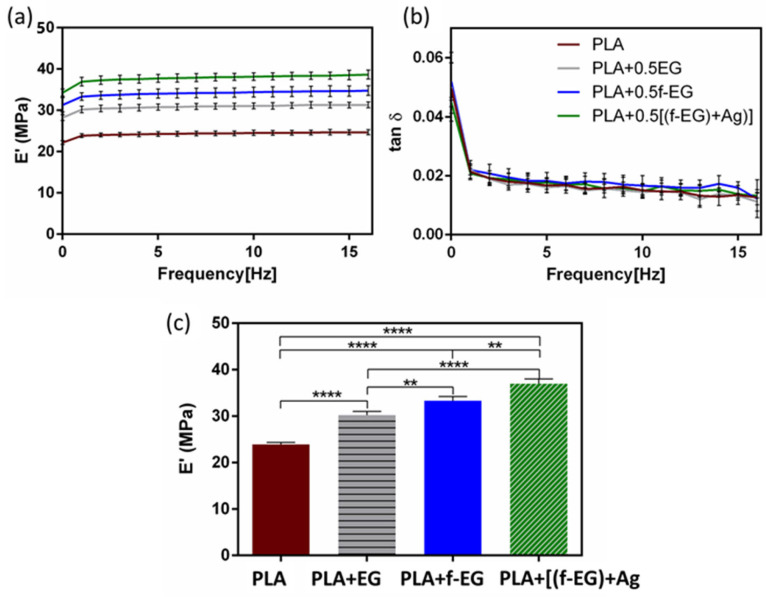
(**a**) Storage modulus and (**b**) the loss factor obtained for 3D-printed PLA scaffolds and scaffolds reinforced with 0.5 wt.% of EG, f-EG, and [(f-EG)+Ag], as a function of the frequency, ranging from 0.01 to 16 Hz. (**c**) Storage modulus of 3D-printed scaffolds at 37 °C and 1 Hz. Significant differences were stated for *p* < 0.01 (**) and *p* < 0.0001 (****).

The mechanical properties of composites based on PLA reinforced with graphene-based materials are often measured under static loading. Composites of PLA and low concentrations of EG/few-layer graphene, produced by melt mixing, were reported to exhibit tensile properties that could be adequate for tendon and ligament regeneration applications, without significantly impairing the ductility [52]. Similar conclusions were obtained in our preliminary work [53] performed on 3D-printed composite scaffolds based on a non-medical-grade PLA reinforced with [f-EG)+Ag]. The resulting stress–strain curves of compression tests as a function of the filler content illustrated that the reinforcement did not significantly affect the ductility, even with a filler content of 2 wt.% [53]. However, ligaments experience dynamic loads during normal locomotion, and their response was influenced by their viscoelastic properties. The viscoelastic properties of the 3D-printed scaffolds of PLA and composites were assessed by using DMA under dynamic conditions after immersion in physiological media overnight, to mimic the physiological conditions. Figure 6a,b show the storage modulus (E’) and the loss factor (tan δ) of the scaffolds as a function of frequency at 37 °C.

For all formulations, the storage modulus slightly increased while the tan δ decreased with increasing frequency, as commonly observed in viscoelastic materials [54,55]. This viscoelastic characteristic was extremely important since native tendons and ligaments also exhibited viscoelastic behavior [7]. No significant differences were found for the damping ability between the PLA and composite scaffolds. The incorporation of EGs in the composites led to an increase in the storage modulus, typical in graphene-reinforced PLA materials [56,57]. At 1 Hz, the PLA scaffolds had the lowest E’ (≃24 MPa). As observed in Figure 6c, for the scaffolds containing PLA+EG and PLA+f-EG, the storage modulus increased approximately 27% and 40%, respectively. Since fillers are stiffer than PLA, that may produce rigid interfaces with PLA and restrict the mobility of polymer chains, resulting in an increased modulus of the composites [56]. The highest increase in the storage modulus was observed for the scaffolds with PLA+[(f-EG)+Ag], which represents an improvement of ≃55% compared with the PLA scaffolds. Here the AgNPs seem to act as a filler increasing the stiffness, as observed by other authors [21,58]. These 3D-printed scaffolds present a storage modulus comparable to values reported in the literature for ligaments/tendons at 1 Hz [54,59], which supports their suitability for the envisaged application. Another strategy to develop scaffolds with a viscoelasticity suitable for ACL regeneration involves the incorporation of a hydrogel in a scaffold, as reported by Freeman et al. [60], which combined 10% of poly(ethylene glycol) (PEG) diacrylate hydrogel with a poly-l-lactic acid scaffold [60].

The incorporation of the different EGs had a significant impact on the viscoelastic/mechanical properties of PLA when compared with other works. For instance, Pinto et al. [61] produced composites of PLA/GNPs (2 wt.%) and PLA/CNTs functionalized with carboxylic acid (0.3 wt.%) by melt mixing/compression molding. The authors observed an increase (4%) in the storage modulus of PLA/CNTs composite relative to the neat PLA matrix, while for the PLA/GNPs composite, they found a decrease of 2%, at 1 Hz and under tension [61]. The existing PLA hybrids/composites for tendon/ligament TE include PLA or poly-l-lactic acid/hydroxyapatite (Hap) [1,16,62], PLA/PEG/Hap [63], PLA/Col [64], or PLA/PLGA [51]. The suggestion of PLA–ceramic composites has been a choice for tissue interfaces between the tendon/ligament and bone [62]. Our scaffolds benefit from the electrically conductive character of (f-EG) and the antibacterial properties of AgNPs, which is a relevant advancement relative to current solutions. The presence of silver nanoparticles also accelerates the tendon healing process, by boosting cell proliferation, and modulates the ECM composition (more and better quality of collagen fibrils) [65].

#### 3.3.3. Biodegradation of Scaffolds

**Figure 7:**
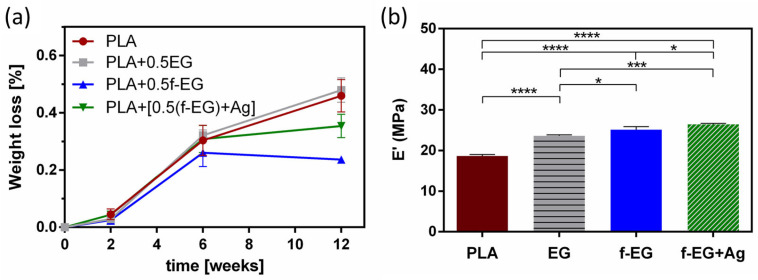
(**a**) Weight loss (%) of 3D-printed scaffolds and (**b**) storage modulus of degraded scaffolds (after 12 weeks), at 37 °C and 1 Hz. Significant differences were stated for *p* < 0.05 (*), *p* < 0.001 (***), and *p* < 0.0001 (****).

A biodegradable scaffold should preserve at least half of its structural and mechanical integrity for a minimum of 3–6 months (for tendon/ligament recovery) and then should degrade gradually [7]. Approximately 6 weeks after the initial injury, remodeling begins and will eventually yield a slightly disorganized ECM [10]. The stability behavior of 3D-printed scaffolds was assessed over a period of 12 weeks and is presented in Figure 7a. After 3 months, a very short reduction from the initial mass (<1%) was verified. Independently of the composition, the degradation was more pronounced between the first 2 and 6 weeks. It has been suggested that during hydrolytic degradation, PLA breaks into lactic acid or into carbon dioxide and water, naturally excreted from the body [66]. Even though there were no statistically significant differences, these results seem to indicate that the incorporation of EG and EG derivatives slightly induced a higher resistance to degradation. This behavior may be related to the nucleation effect induced by EGs, increasing the crystallization of the polymer [61]. Pinto et al. [61] also assessed the hydrolytic degradation of composites based on PLA and (1 wt.%) GNPs over 16 weeks and found a maximum weight loss of 5% and a comparable behavior of PLA and its composites [61]. By analyzing Figure S6, it is possible to compare the surface images of non-degraded and degraded (after 12 weeks) 3D-printed scaffolds that illustrate these conclusions. After 3 months of hydrolytic degradation, some surface erosion and the existence of pores on the surface of degraded scaffolds were visible, being more pronounced in neat PLA and PLA+0.5EG scaffolds. In addition, as expected, the storage modulus of the degraded samples at 37 °C and 1 Hz decreased when compared with non-degraded samples, remaining in the same order of magnitude (MPa) (Figure 7b).

#### 3.3.4. Biological Assays

**Figure 8:**
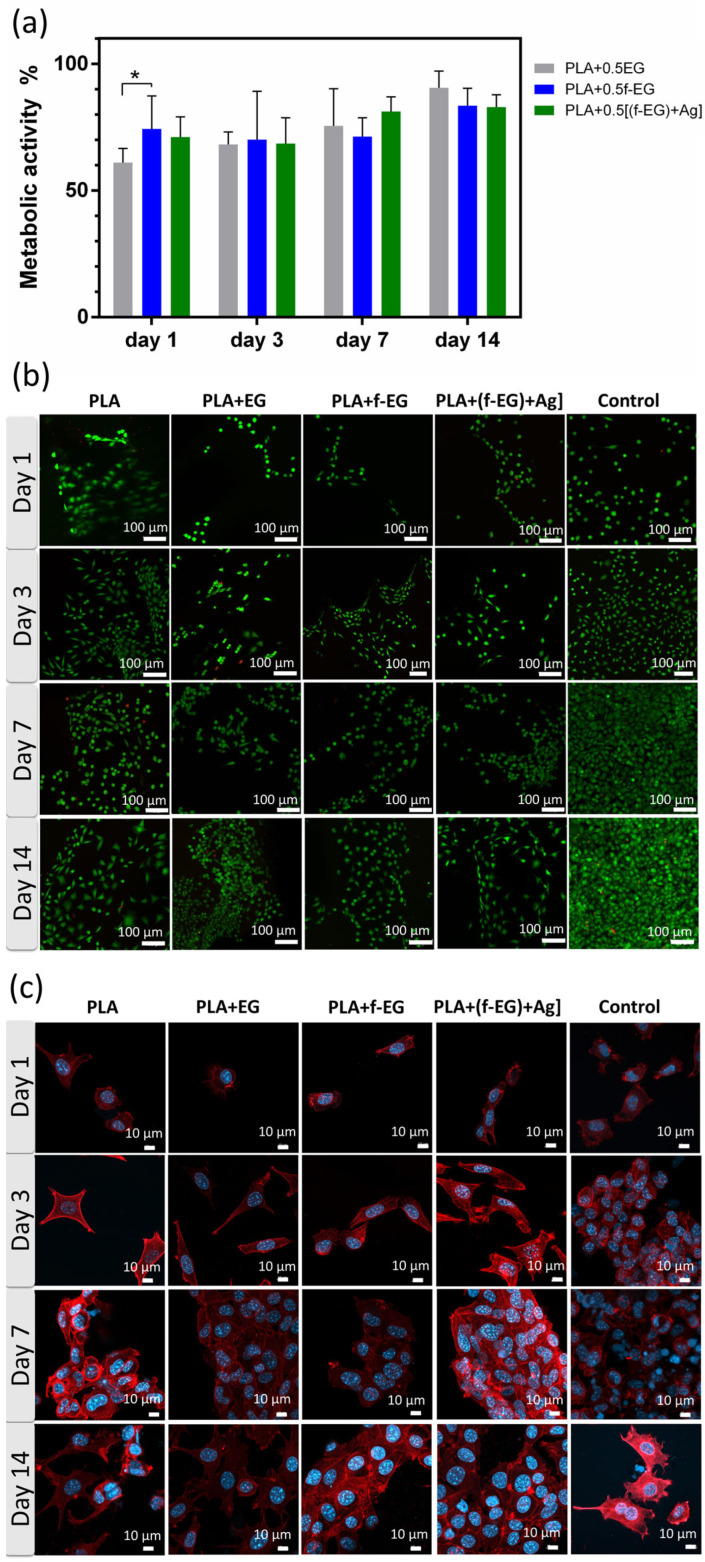
*In vitro* cell culture on the scaffolds. (**a**) Metabolic activity of L929 cells determined by Alamar blue cell viability assay. Data normalized to PLA (100% metabolic activity). Significant differences, effect of the material: *p* < 0.05 (*). (**b**) Representative fluorescent images of live (green)/dead (red) cells seeded on the scaffolds. (**c**) Fluorescence image of L929 cells seeded on 3D-printed scaffolds and TCPS. Cells were immunostained for F-actin with phalloidin (red), and cell nuclei were stained with DAPI (blue).

**L929.** The cytocompatibility of the produced scaffolds was evaluated through the *in vitro* culture of L929 fibroblast cells in direct contact with the scaffolds. The scaffolds must be able to withstand sterilization without physical, chemical, and biological change. Their metabolic activity and viability were assessed after 1, 3, 7, and 14 days of culture (Figure 8a,b). The L929 morphology and cytoskeleton organization were investigated by using SEM (Figure S7) and a DAPI-phalloidin test (Figure 8c).

As observed in Figure 8a, at early stages of culture (days 1 and 3) the metabolic activity of cells on composites exhibited values around 70% relative to those of PLA. After 14 days, their metabolic activity increased and became similar to that of the PLA scaffolds, with the cells on PLA+EG scaffolds exhibiting the highest value (≃91%), followed by PLA+ f-EG (≃84%) and PLA+[(f-EG)+Ag] (≃83%). As illustrated in the live/dead images (Figure 8b), none or almost none dead cells (red) were found in composite scaffolds. Such features suggest that the addition of EG and EG derivatives did not affect the metabolic activity of the fibroblasts or their cellular viability, discarding the potential toxic effects of EGs. Thus, all studied scaffolds can be considered non-cytotoxic. Other authors also obtained biocompatible composites of PLA reinforced with a low content (0.4 wt.%) of GNPs or GO [18,27]. In a period of 3 days of culture, Gonçalves et al. [18] also found high metabolic activity of fibroblasts in PLA–GNPs, never below 97% when compared with PLA [18].

After 24 h of incubation, cells adhered well on 3D-printed scaffolds, although presenting a rounder shape in the composite scaffolds than on the PLA (Figure 8c). Fine filopodial extensions were also visible. On the third day, an elongated spindle-like morphology was observed for all compositions, with higher surface attachment than on the control. A higher increase in the cell density was observed at the 7th up to the 14th day, reaching confluency for all studied conditions. The proliferation was even more pronounced than on the control, with the cells covering large areas of the scaffolds’ surfaces. The nanoroughness caused by FDM and by the presence of nanoparticles increased the surface area, and this was shown to positively influence the attachment, migration, and orientation of various cell lines including fibroblasts, which play a critical role in the healing process [11,27].

**Figure 9:**
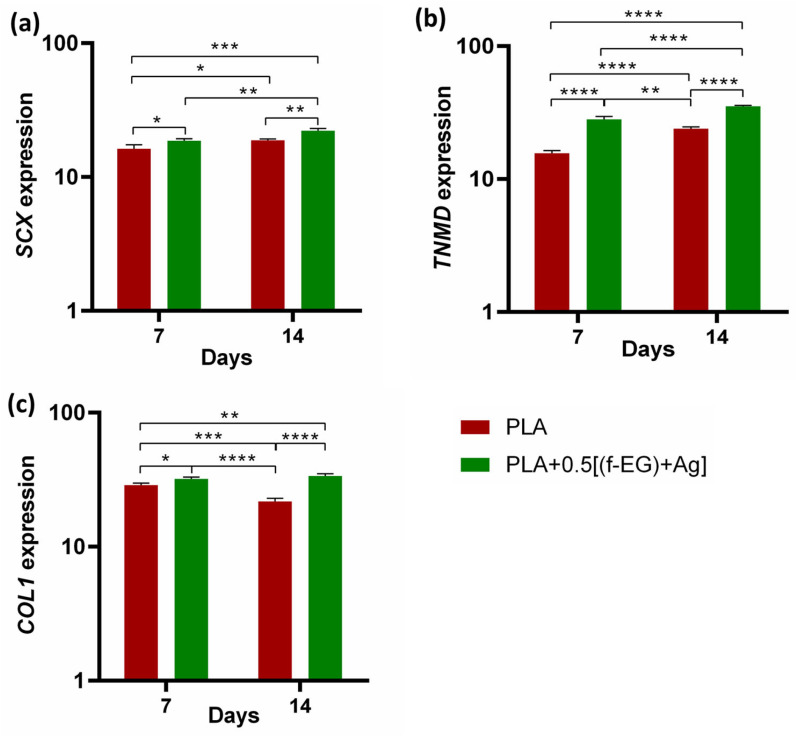
Assessment of the genetic expression of tenogenic markers of hTDCs in 3D scaffolds after 7 and 14 days of culture. Relative gene expression of (**a**) *SCX*, (**b**) *TNMD*, and (**c**) *COL1*. Symbols denote statistical differences: * for *p* < 0.05, ** for *p* < 0.01, *** for *p* < 0.001, and **** for *p* < 0.0001.

**Human tendon-derived cells in PLA and PLA+0.5[(f-EG)+Ag] scaffolds—Expression of tendon-related markers.** Due to the similarities among tissues and to the expression of common markers such as TNMD and SCX [31,67], engineering tendons and ligaments have been pursued with common strategies. In this work, the potential of PLA+0.5[(f-EG)+Ag] scaffolds to support the tenogenic/ligamentogenic phenotype was assessed in hTDCs through gene expression analysis (Figure 9). The resident cell populations including stem/progenitor cells subsets have an epigenetic commitment to respond to tendon and ligament-specific requirements and a natural role in the renewal and maintenance and of tissue composition and properties, with impact on the healing process and its outcomes [31,67]. These cells exhibit clonogenicity, self-renewal, and multipotency, as well as a high expression of scleraxis (SCX), tenomodulin (TNMD), and collagen type I (COL1) [31].

**Figure 10:**
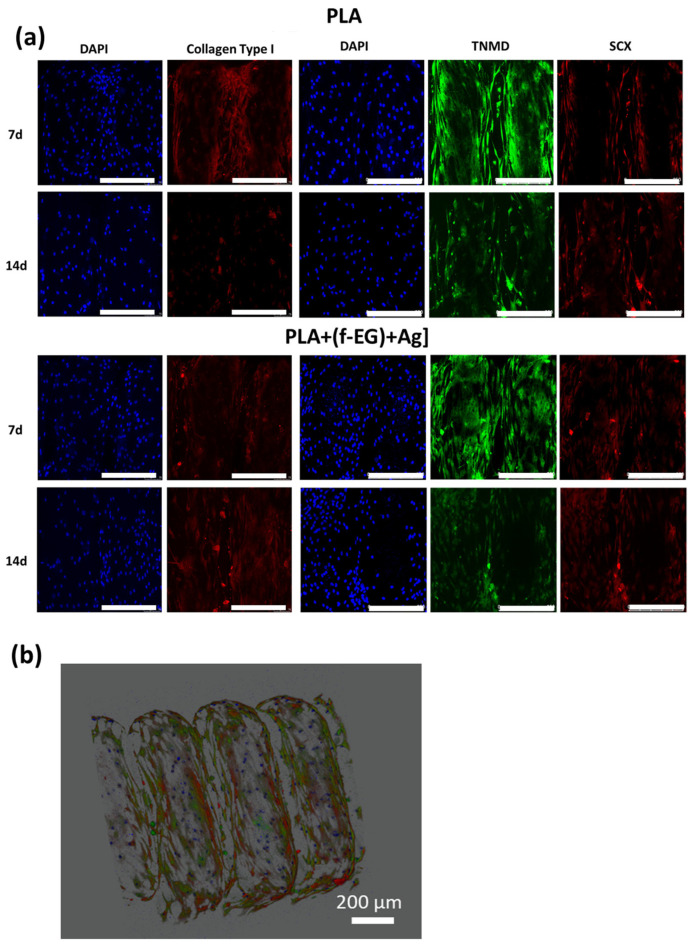
Immune-location of (**a**) COL1 (red), SCX (red), and TNMD (green) in hTDCs-laden scaffolds after 7 and 14 days of culture (20×, scale bar 250 μm). Nuclei were stained with DAPI (blue). (**b**) A 3D image reconstruction of the z-stacks collected from the hTDCs-laden PLA+[0.5(f-EG)+Ag] scaffold, evidencing scaffold structure and distribution of TNMD (green) and SCX (red) produced by hTDCs (nuclei, blue).

Cells cultured on both types of scaffolds showed increased expression of tendon/ligament markers, namely, *SCX*, *TNMD*, and *COL1*, during the experimental setup. *SCX, TNMD*, and *COL1* evidenced an upregulation from day 7 to day 14, suggesting that the 3D-printed scaffolds assisted the maintenance of the hTDCs’ phenotype [68]. In addition, the gene expression levels of SCX, TNMD, and COL1 were significantly increased by the presence of (f-EG)+Ag, suggesting that the composite scaffolds supported the tenogenic phenotype [30]. In accordance, the expressions of *SCX* and *COL1* were significantly increased in hTDCs cultured on the composites (*SCX*: at day 7, *p* < 0.05, and at day 14, *p* < 0.01, and *COL1*: at day 7, *p* < 0.05, and at day 14, *p* < 0.0001), relative to hTDCs on the PLA scaffolds. The levels of TNMD showed a significant increase in composite scaffolds when compared with PLA, at each and every time point (*p* < 0.0001). We further investigated the locations of the tendon-related proteins in the scaffolds, which were analyzed by immunofluorescence (Figure 10a,b). SCX, TNMD, and COL1 were observed over time in cells cultured on both types of scaffolds. The cells were homogenously distributed, evidencing a dense colonization of the scaffolds’ structures. Moreover, seeded cells exhibit a fusiform morphology and a tendency to organize themselves into parallel alignment on the surface of the scaffolds, which is typically observed in tendon cells in native tissues. The obtained results support that 3D-printed scaffolds encourage the colonization of hTDCs and the expression of genes and proteins associated to the tenogenic/ligamentogenic phenotype and, therefore, hold the potential to sustain healing strategies aiming to regenerate tendons and ligaments. Similar results were found for other graphene-based polymer scaffolds [69,70]. These are encouraging results for further *in vivo* experiments.

The 3D-printed scaffold developed could be further explored both as an acellular and cell-laden scaffold. As a promising tendon and ligament TE product, the inclusion of cells as a component introduces risks, and for that reason the selection for an acellular scaffold as medical device should be less time-consuming and face less regulatory scrutiny. In European Union (EU), medical devices are strictly regulated both by national competent authorities and by the European Medicines Agency (EMA), while in the USA the extensive regulatory requirements are defined by the U.S. Food and Drug Administration (FDA). 

## 4. Conclusions and Future Work

The 3D-printed scaffolds based on medical-grade PLA reinforced with 0.5 wt.% of EG, f-EG, and (f-EG)+Ag were successfully produced and characterized. EG was organically functionalized and decorated with silver nanoparticles. The aim of the organic functionalization of EG was to strengthen the interface with the polymer and to provide anchoring sites for Ag, allowing the inclusion of a small concentration of Ag. The Ag anchored on EG acted as an anti-microbial agent, as confirmed against microorganisms of clinical relevance *S. aureus* and *E. coli*, an important feature for the healing process and prevention of bacterial infections. PLA composite filaments were melt-extruded with a good filler dispersion and used for the fabrication of customized 3D porous scaffolds. Highly reproducible scaffolds were obtained with a porosity of 64–71% and a network of interconnected pores of around 400 μm. The scaffolds’ biodegradation and mechanical properties were evaluated. All scaffolds exhibited high stability and surface nanoroughness, which was increased by the fillers’ presence. The wet state dynamic mechanical analysis proved that the addition of reinforcements led to a significant increase in the storage modulus, being mechanically adequate for tendon and ligament applications. The highest increase was observed for scaffolds with PLA+0.5[(f-EG)+Ag], which represents an increase of ≃55% compared with PLA scaffolds. Similar to native living tissues, the scaffolds exhibited a viscoelastic behavior. The PLA+[(f-EG)+Ag] scaffolds were non-toxic and showed capability to maintain the tenogenic commitment of human tendon-derived cells, with an increase in the gene expression of specific tendon/ligament-related markers. The results demonstrate the possibility for easy, cost-effective, and personalized 3D-printed scaffolds with great potential applications for tendon and ligament regeneration. We believe that this article presents compelling *in vitro* results for further *in vivo* experiments. Some strategies may be considered for future directions of this research, to improve the scaffolds’ overall performance and clinical applications, namely, their capability to inhibit mycobacteria [71] or even adapt and change over time [72].

## Figures and Tables

**Table 1:** Printing settings used for printing PLA and PLA composite scaffolds.

Printing Parameters	
Nozzle diameter	0.4 mm
Nozzle temperature	190 °C
Bed temperature	80 °C
Printing speed	45 mm.s^−1^
Layer height	0.15 mm
Infill distance	0.8 mm
Infill density	50%
Infill pattern	Lines
Infill lines direction	0°; 90°
Support contact angle	5°

**Table 2:** Presence (+) or absence (−) of *E. coli* and *S. aureus* at concentrations (0.025–1%) of different EGs.

	Concentration (%)	*E. coli*	*S. aureus*
**EG**	1	+	+
	0.5	+	+
	0.25	+	+
	0.1	+	+
	0.05	+	+
	0.025	+	+
**f-EG**	1	+	+
	0.5	+	+
	0.25	+	+
	0.1	+	+
	0.05	+	+
	0.025	+	+
**(f-EG)+Ag**	1	−	−
	0.5	−	−
	0.25	−	−
	0.1	−	+
	0.05	+	+
	0.025	+	+

## Data Availability

Data are contained within the article and Supplementary Materials.

## Supplementary Materials

The following supporting information can be downloaded at https://www.mdpi.com/article/10.3390/nano13182518/s1: Table S1: Operating parameters used for the production of composite filaments. Table S2: Primers used for real-time quantitative RT-PCR analysis. Figure S1: Minimum bactericidal concentration of different EGs (EG, f-EG, and (f-EG)+Ag) against *E.coli* and *S. aureus*. Figure S2: SEM images of (a) PLA pellet and PLA pellet coated with 0.5 wt.% of (b) pristine EG, (c) f-EG, and (d) (f-EG)+Ag powder. The insets represent different magnifications. Figure S3: SEM images of filaments’ cross-sections: (a) PLA, (b) PLA+0.5EG, (c) PLA+0.5f-EG, and (d) PLA+0.5[(f-EG)+Ag]. (e) MFI values of filaments. Table S3: Mean pore size of the 3D-printed scaffolds. Figure S4: SEM images of the cross-section of 3D-printed scaffolds formed by (a) PLA, (b) PLA+0.5EG, (c) PLA+0.5f-EG, and (d) PLA+0.5[(f-EG)+Ag]. Figure S5: EDS of PLA+0.5[(f-EG)+Ag] scaffolds. Figure S6: SEM images of the surface of (a,a1) PLA, (b,b1) PLA+0.5EG, (c,c1) PLA+0.5f-EG, and (d,d1) PLA+0.5(f-EG)+Ag scaffolds at stage 0 and after 12 weeks of degradation, respectively. Figure S7: SEM images of the L929 cells seeded on PLA, PLA+EG, PLA+f-EG, and PLA+[(f-EG)+Ag] scaffolds, after 1, 3, 7, and 14 days. Magnifications for closer observation of L929 cells.
