# Supplementary material for: Biocompatible 3D-Printed Tendon/Ligament Scaffolds Based on Polylactic Acid/Graphite Nanoplatelet Composites
Source: Nanomaterials (Basel). 2023 Sep 8;13(18):2518. doi: 10.3390/nano13182518 (PMC10536374; doi:10.3390/nano13182518)
Supplement: Supplementary file 1 [file nanomaterials-13-02518-s001.zip › nanomaterials-2556937-supplementary.pdf]

Table S1. Operating parameters used for the production of composite filaments.

Filament	Feed/Barrel/Die Temperature (°C)	Feed Rate ($g.min^{-1}$)	Pulling Rolls R1($mm.s^{-1}$)	Pulling Rolls R2($mm.s^{-1}$)
**PLA**	135-185-170	4.2	27.84	31.64
**PLA+0.5EG**	135-185-168		45.70	47.87
**PLA+0.5f-EG**	135-185-163		34.50	35.50
**PLA+0.5[(f-EG)+Ag]**	135-185-155		32.81	35.85

Table S2. Primers used for real-time quantitative RT-PCR analysis.

	Primer Sequence	Accession Number
**Human Glyceraldehyde 3-phosphate dehydrogenase (*GAPDH*)**	F - TGTACCACCAACTGCTTAGC<br>R - GGCATGGACTGTGGTCATGAG	NM_002046.4
**Scleraxis (SCXA)**	F – CGAGAACACCCAGCCCAAAC<br>R – CTCCGAATCGCAGTCTTTCTGTC	XM_001717912
**Collagen, Type I, alpha 1 (COL1A1)**	F – CGAAGACATCCCACCAATCAC<br>R – GTCACAGATCACGTCATCGC	NM_000088.3
**Tenomodulin (TNMD)**	F – CCGCGTCTGTGAACCTTTAC<br>R – CACCCACCAGTTACAAGGCA	NM_022144.2

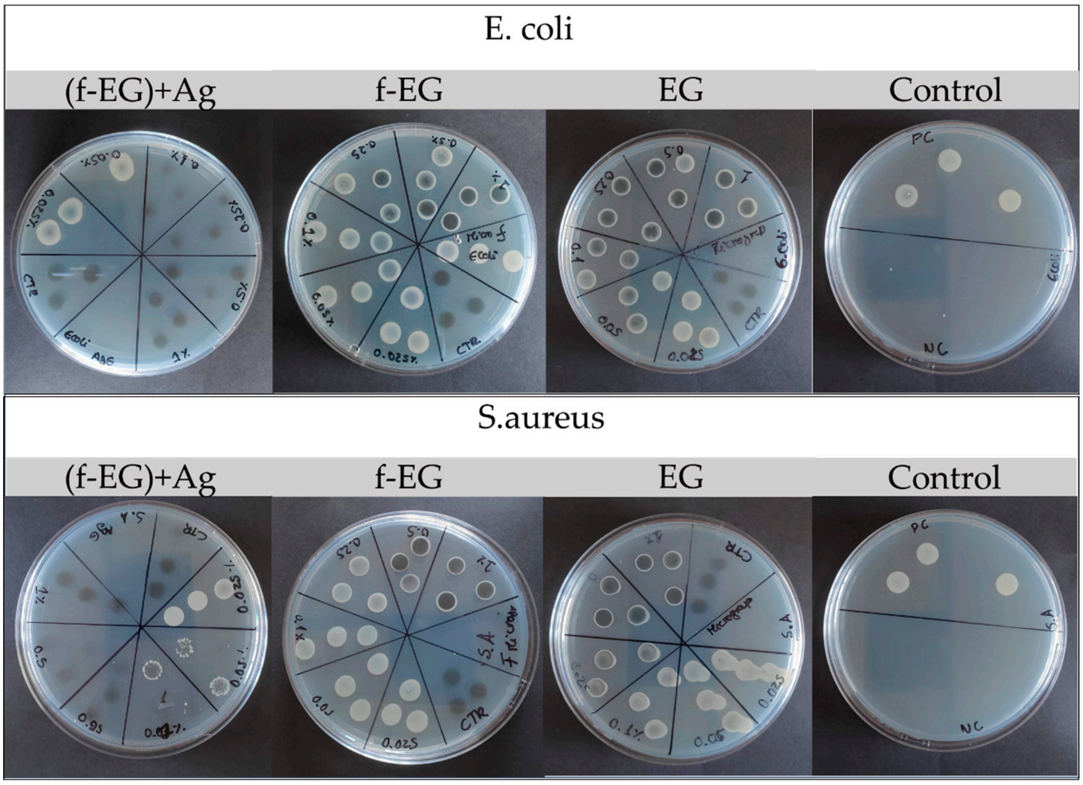

Figure S1. Minimum bactericidal concentration of different EGs (EG, f-EG, and (f-EG)+Ag against *E.coli* and *S. aureus*.

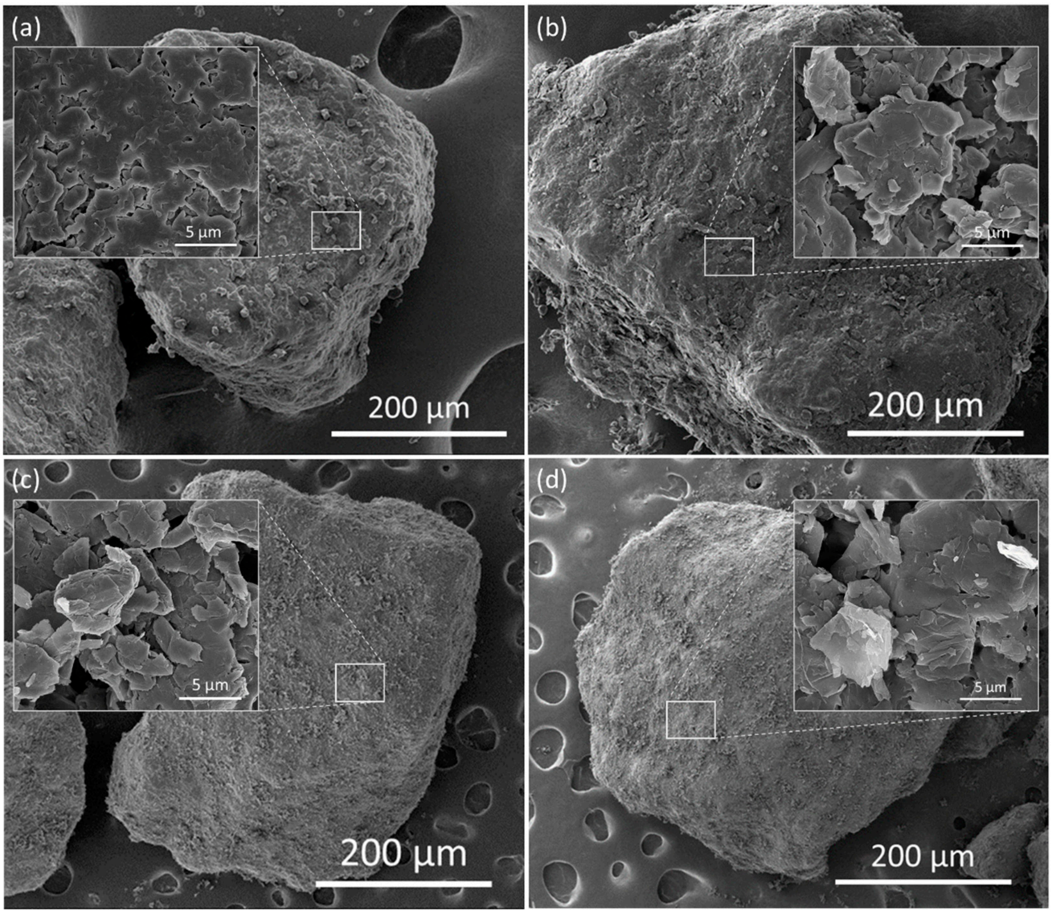

Figure S2. SEM images of (**a**) PLA pellet and PLA pellet coated with 0.5 wt.% of (**b**) pristine EG, (**c**) f-EG, and (**d**) (f-EG)+Ag powder. The insets represent different magnifications.

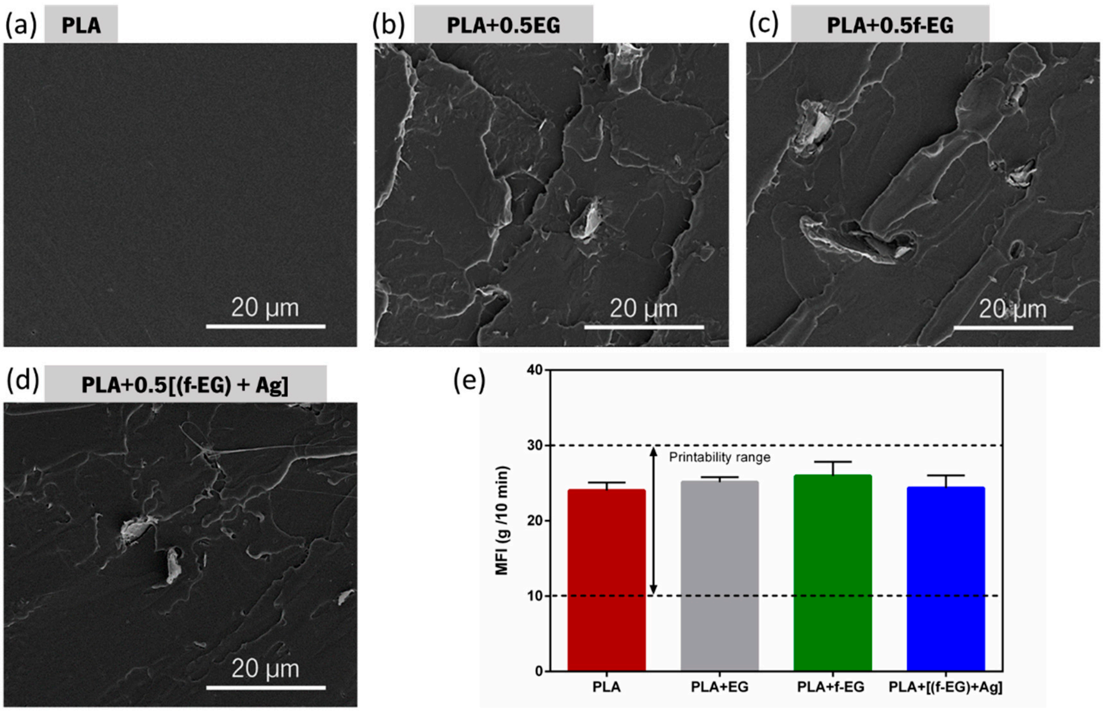

Figure S3. SEM images of filaments' cross-sections: (**a**) PLA, (**b**) PLA+0.5EG, (**c**) PLA+0.5f-EG, and (**d**) PLA+0.5[(f-EG)+Ag]; (**e**) MFI values of filaments.

Table S3. Mean pore size of the 3D-printed scaffolds.

Scaffold		Pore Size (mm)
**PLA**		0.43 ± 0.13
**PLA+0.5**	EG	0.45 ± 0.11
**PLA+0.5**	f-EG	0.42 ± 0.13
**PLA+0.5**	[(f-EG)+Ag]	0.43 ± 0.10

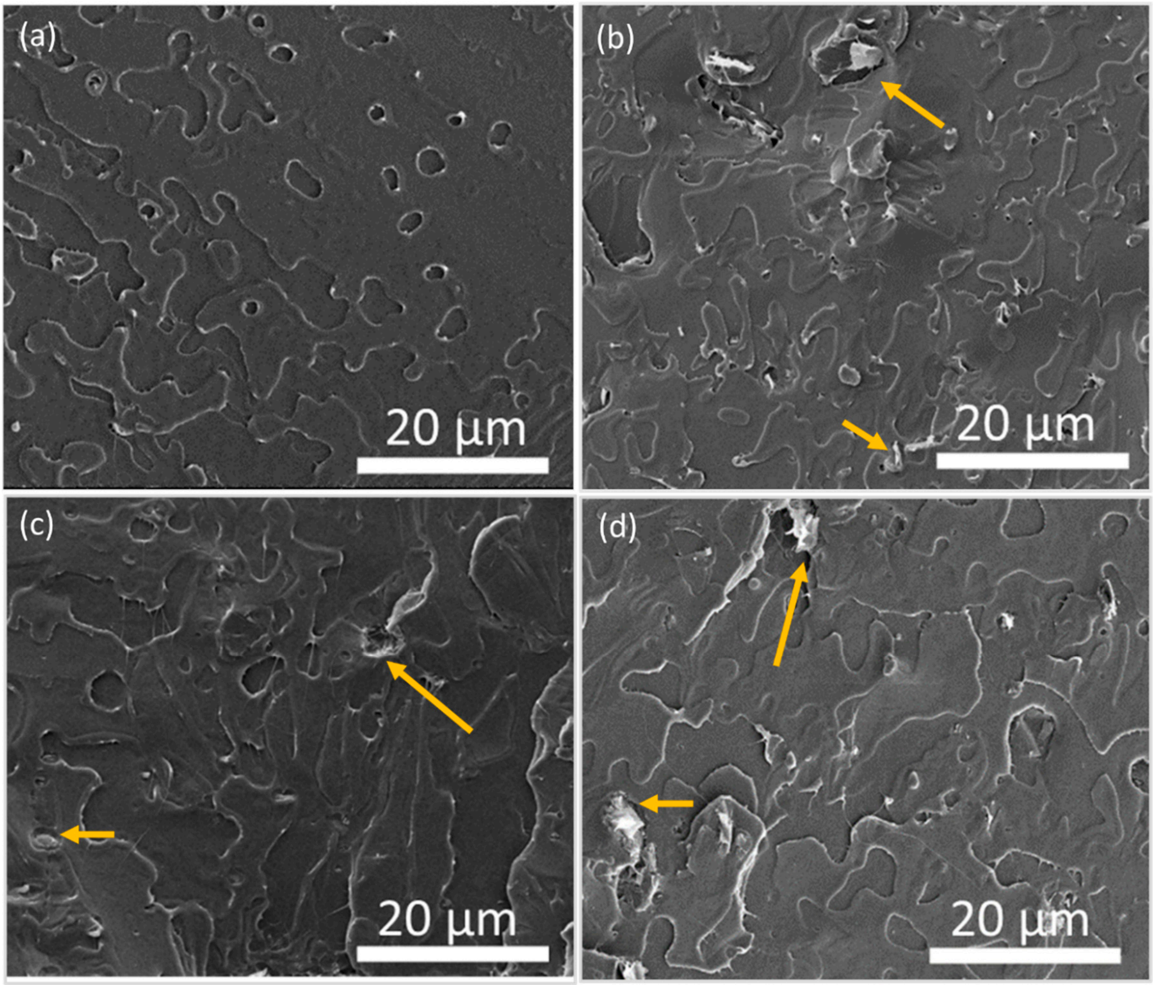

Figure S4. SEM images of the cross-section of 3D printed scaffolds formed by (**a**) PLA, (**b**) PLA+0.5EG, (**c**) PLA+0.5f-EG, and (**d**) PLA+0.5[(f-EG)+Ag].

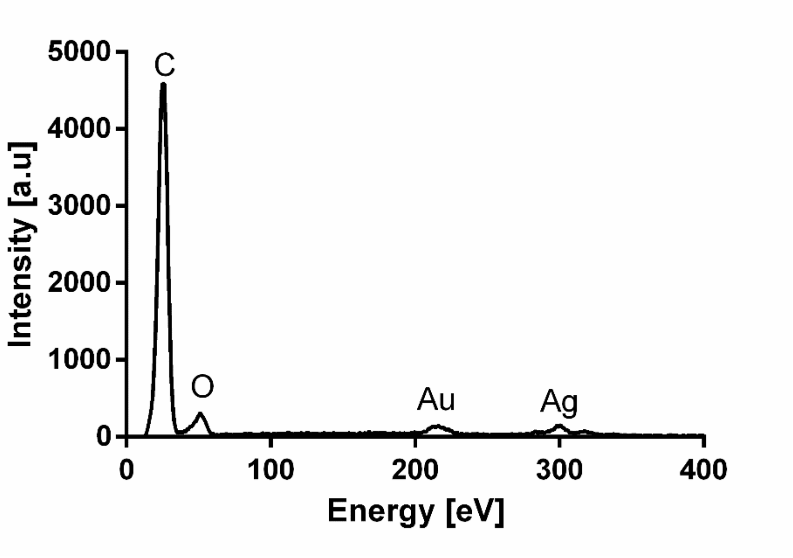

Figure S5. EDS of PLA+0.5[(f-EG)+Ag] scaffolds.

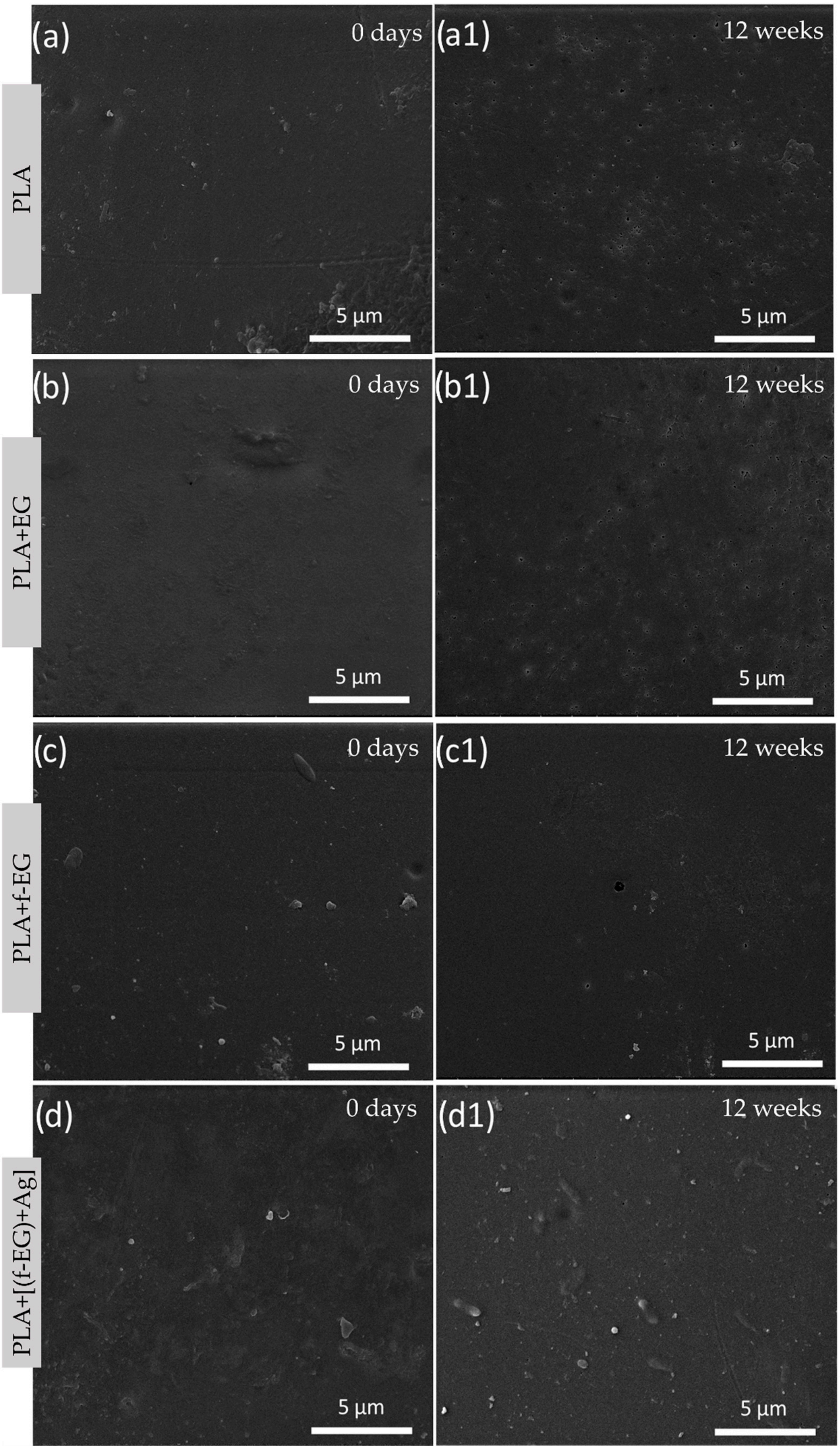

Figure S6. SEM images of the surface of (**a**,**a1**) PLA, (**b**,**b1**) PLA+0.5EG, (**c**,**c1**) PLA+0.5f-EG, and (**d**,**d1**)PLA+0.5(f-EG)+Ag scaffolds at stage 0 and after 12 weeks of degradation, respectively.

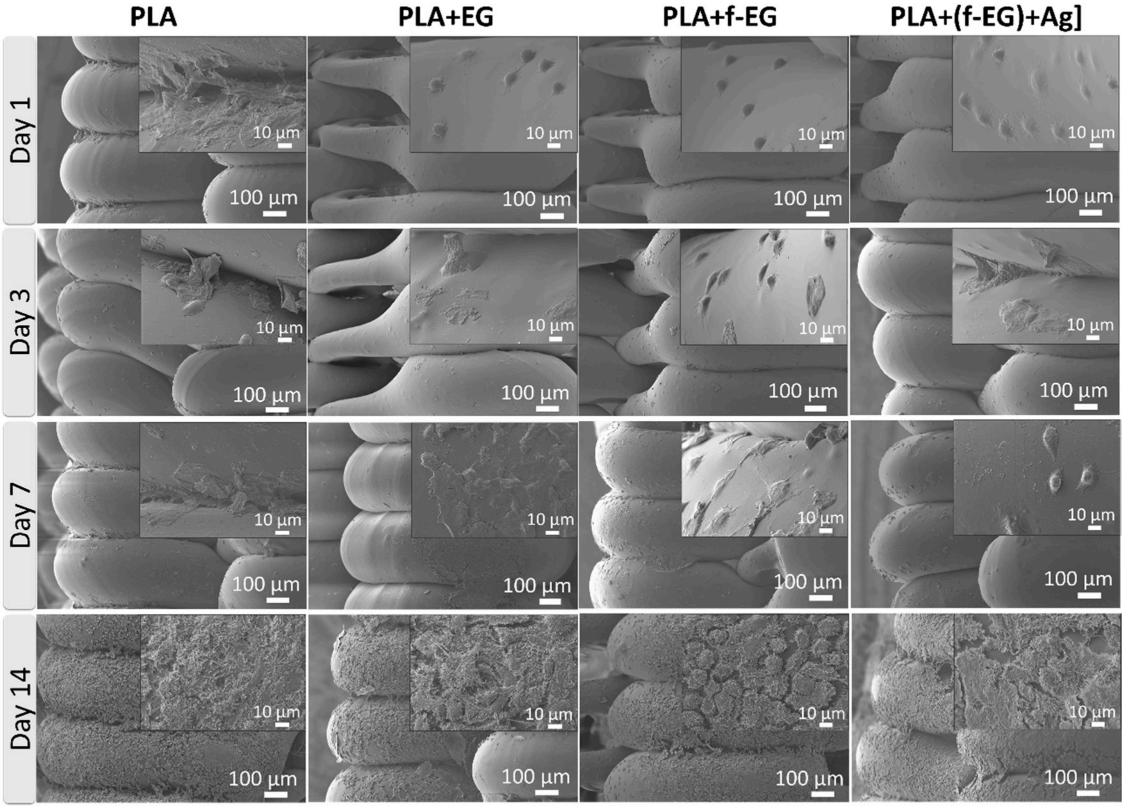

Figure S7. SEM images of the L929 cells seeded on PLA, PLA+EG, PLA+f-EG and PLA+[(f-EG)+Ag] scaffolds, after 1, 3, 7, and 14 days. Magnifications for closer observation of L929 cells.
